# Supplementary material for: Global loss of DNA methylation uncovers intronic enhancers in genes showing expression changes
Source: Genome Biol. 2014 Sep 20;15(9):469. doi: 10.1186/s13059-014-0469-0 (PMC4203885; doi:10.1186/s13059-014-0469-0)
Supplement: Additional file 1: — Sequencing and peak metrics for all ChIP-seq, RNA-seq, and WGBS datasets. ChIP-seq reads were mapped using BWA and peaks were called using Sole-Search [37,38]. All data was collected as part of this study except for H3K4me3 from HCT116, which was from ENCODE. However, for consistency, the reads were remapped and peaks were called for this dataset using Sole-Search. High-confidence (HC) peaks were determined as those present in two independent biological replicates. Promoter-proximal or promoter-distal peaks were selected by their proximity to a TSS, with proximal being those peaks within 2000 bp of a TSS and distal being everything else. [file 13059_2014_469_MOESM1_ESM.docx]

**Table 1:** **Sequencing and peak metrics for histone and Pol2 ChIP-seq data in HCT116 and DKO1**

|  | H3K4me3 HCT116 | H3K4me3 DKO1 | H3K27ac HCT116 | H3K27ac  DKO1 | RNAPII HCT116 | RNAPII DKO1 |
| --- | --- | --- | --- | --- | --- | --- |
| Unique reads, Rep1 | 17,186,937 | 24,698,737 | 11,448,742 | 19,797,796 | 41,786,921 | 44,000,451 |
| SoleSearch peaks, Rep1 | 18,847 | 17,192 | 29,756 | 22,777 | 25,068 | 13,420 |
| Median peak tag height Rep1 | 65 | 33 | 13 | 15 | 58 | 28 |
| Unique reads, Rep2 | 20,181,661 | 30,120,817 | 18,585,678 | 38,326,644 | 14,961,748 | 27,558,447 |
| SoleSearch peaks, Rep2 | 19,139 | 61,948 | 28,077 | 25,514 | 18,194 | 7,001 |
| Median peak tag height, Rep2 | 63 | 11 | 16 | 15 | 28 | 28 |
| HC peaks | 18,616 | 16,869 | 23,911 | 20,300 | 16,806 | 6,853 |
| Median peak tag height | 69 | 34 | 18 | 17 | 112 | 33 |
| HC proximal or distal peaks | 12,483 (proximal) | 12,817 (proximal) | 10,362 (proximal)  9,183 (distal) | 9,015 (proximal)  10,537 (distal) | 12,004 (proximal) | 5,795 (proximal) |
| Median HC proximal or distal peak height | 96 (proximal) | 41 (proximal) | 27 (proximal)  11 (distal) | 18 (proximal)  16 (distal) | 122  (proximal) | 33  (proximal) |
