# Supplementary figures and images for: Global loss of DNA methylation uncovers intronic enhancers in genes showing expression changes
Source: Genome Biol. 2014 Sep 20;15(9):469. doi: 10.1186/s13059-014-0469-0 (PMC4203885; doi:10.1186/s13059-014-0469-0)

# H3K4me3 proximal peaks

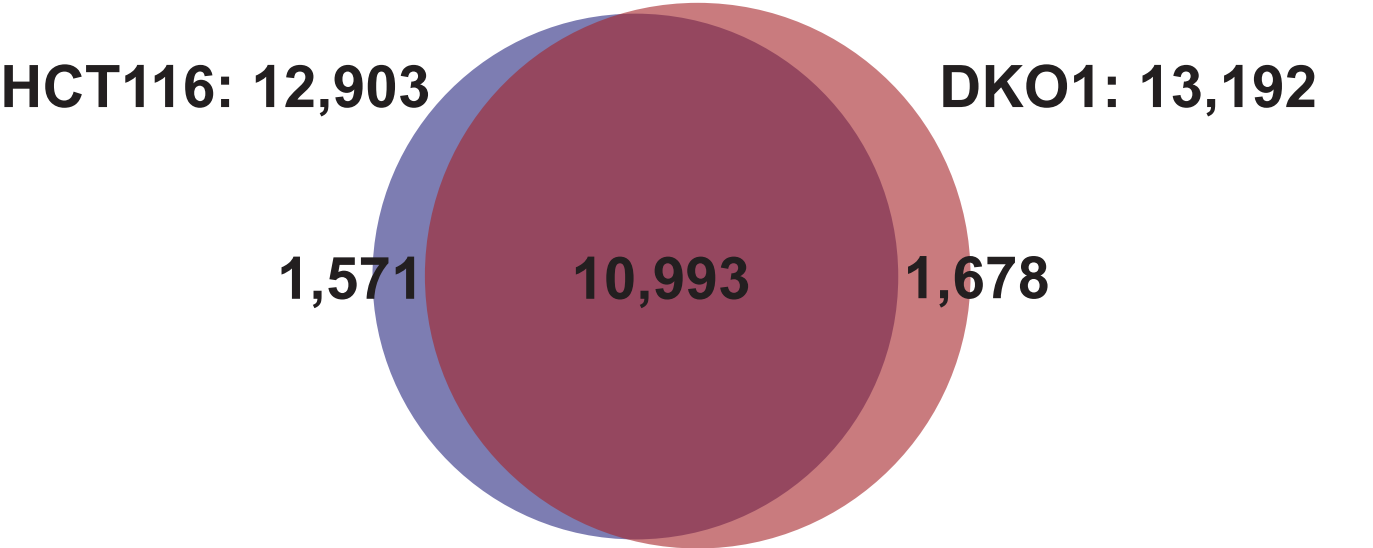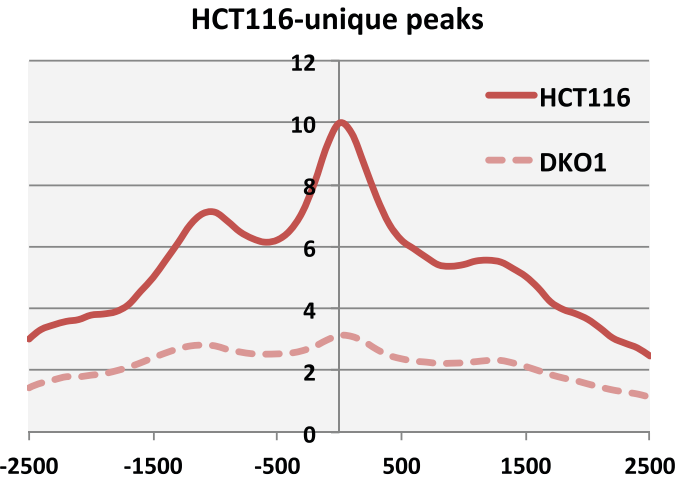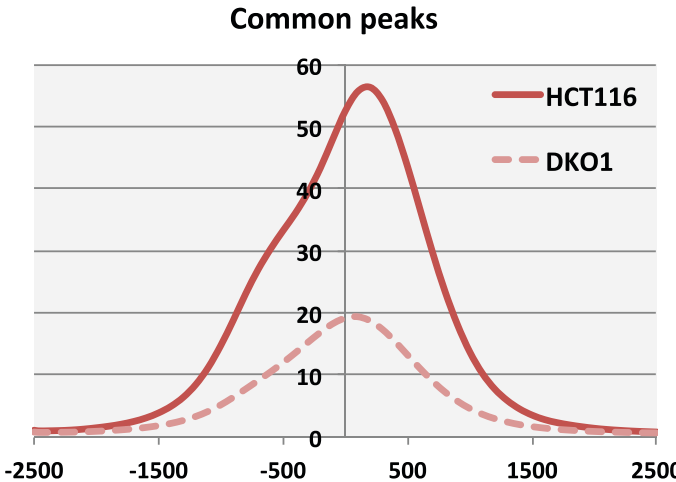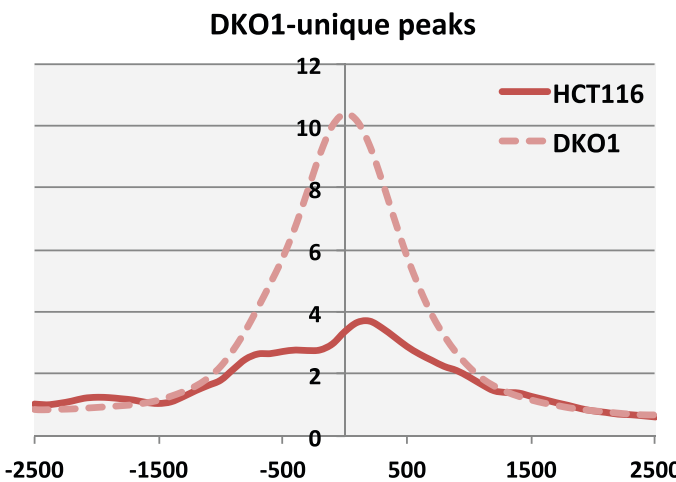

Supplement: Additional file 4: — Characterization of H3K4me3 promoter-proximal peaks in HCT116 and DKO1 cells. Venn diagram showing differences in binding sites for promoter-proximal H3K4me3 peaks (top), and the density of H3K4me3 ChIP-seq tags in HCT116 and DKO1 for all three peak categories (bottom). [file 13059_2014_469_MOESM4_ESM.pdf]

# H3K27ac proximal peaks

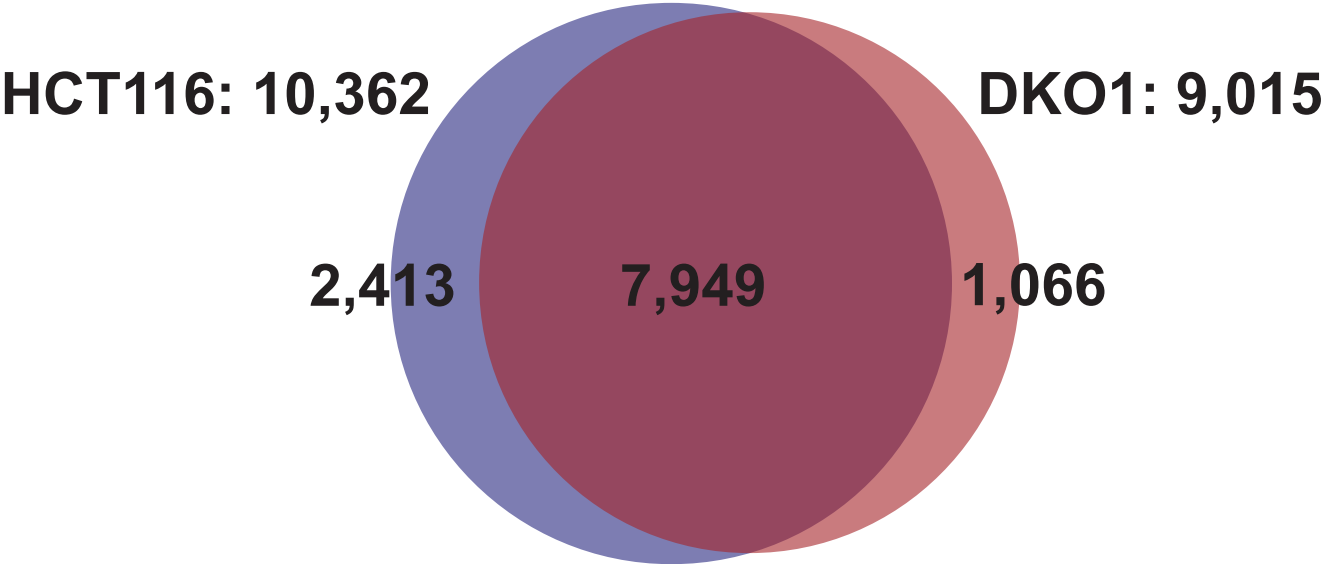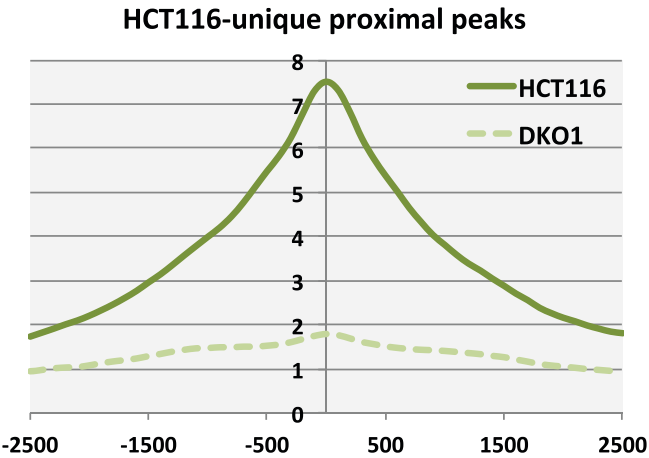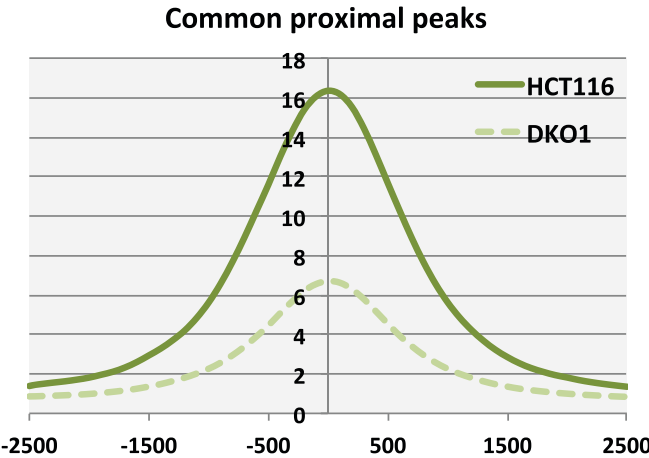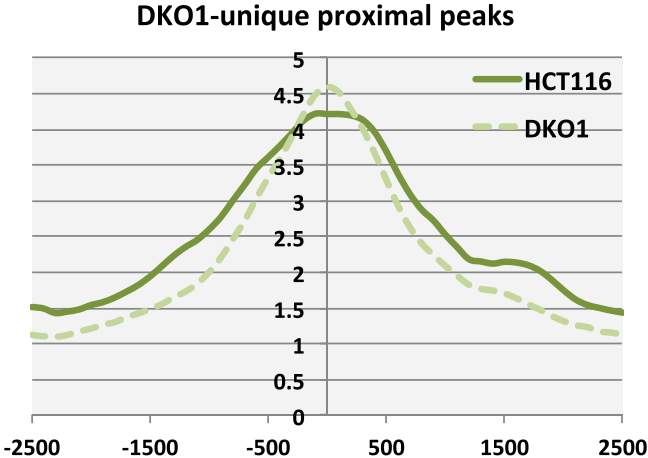

Supplement: Additional file 5: — Characterization of H3K27ac promoter-proximal peaks in HCT116 and DKO1 cells. Venn diagram showing differences in binding sites for promoter-proximal H3K27ac peaks (top), and the density of H3K27ac ChIP-seq tags in HCT116 and DKO1 for all three peak categories (bottom). [file 13059_2014_469_MOESM5_ESM.pdf]

# Analysis of RNA-seq replicates

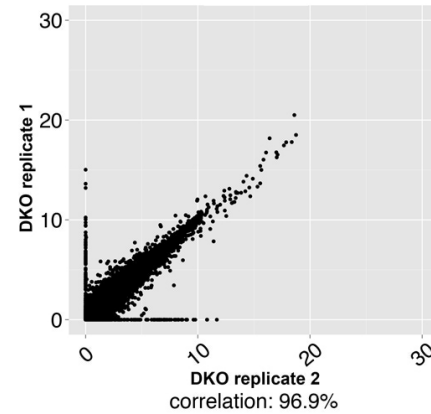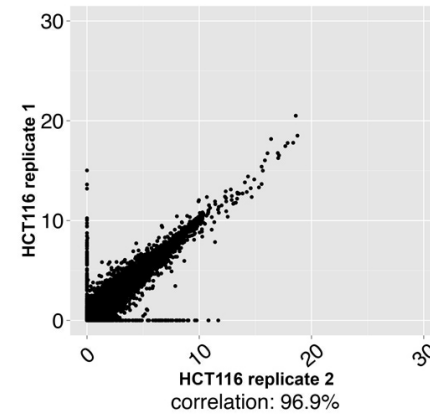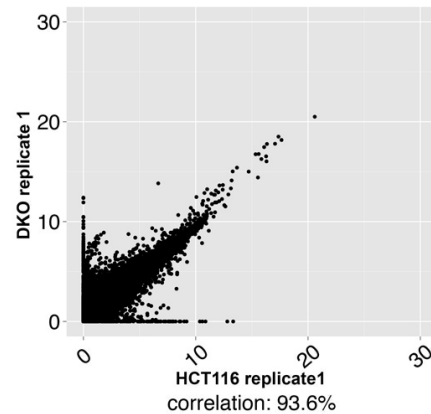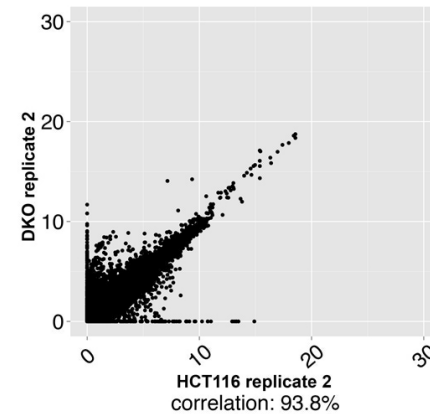

Supplement: Additional file 7: — Replicate RNA-seq analysis. log2 expression values are plotted comparing replicates for of the same cell type, and across cell types for HCT116 and DKO1 cells. [file 13059_2014_469_MOESM7_ESM.pdf]
