# Supplementary material for: Global loss of DNA methylation uncovers intronic enhancers in genes showing expression changes
Source: Genome Biol. 2014 Sep 20;15(9):469. doi: 10.1186/s13059-014-0469-0 (PMC4203885; doi:10.1186/s13059-014-0469-0)

Additional File 6

RNAPII proximal peaks

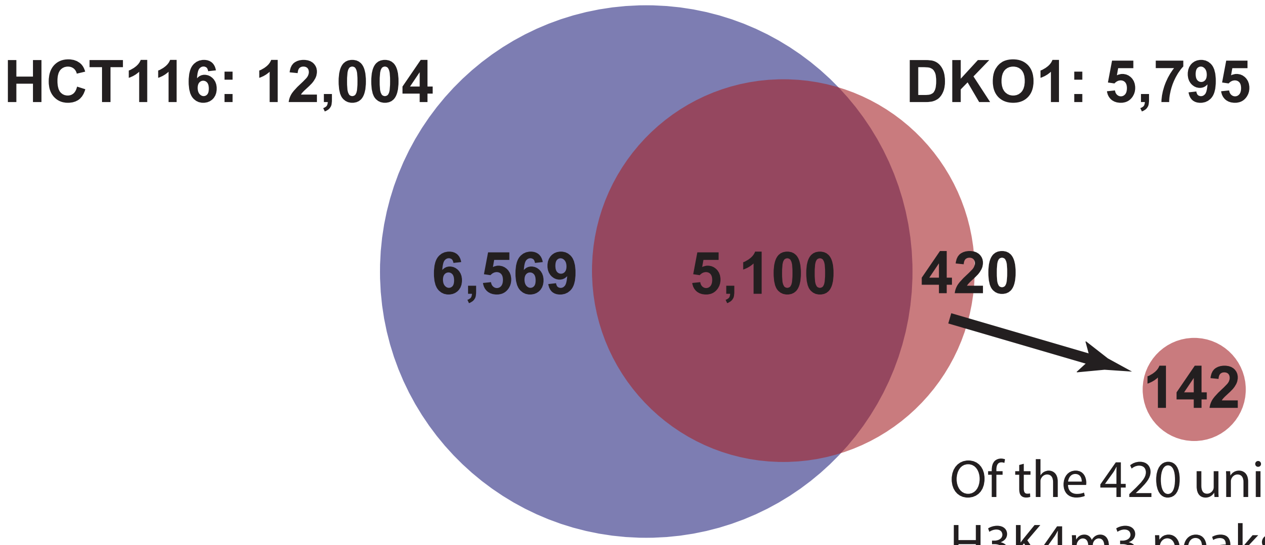

Of the 420 uniquely-called H3K4m3 peaks in DKO1, only 142 had a higher enrichment in DKO1 (shown below)

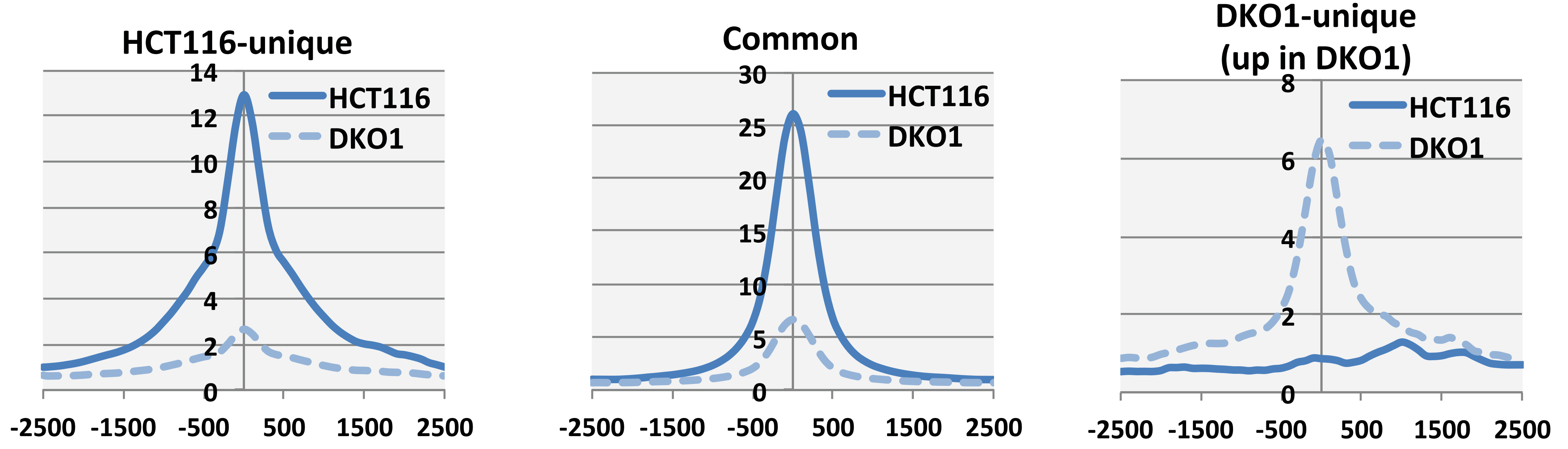

Supplement: Additional file 6: — Characterization of RNAP2 promoter-proximal peaks in HCT116 and DKO1 cells. Venn diagram showing differences in binding sites for promoter-proximal RNA polymerase 2 peaks (top), and the density of RNA polymerase 2 ChIP-seq tags in HCT116 and DKO1 for all three peak categories (bottom). [file 13059_2014_469_MOESM6_ESM.pdf]
